# Pathogenesis of PM_2.5_-Related Disorders in Different Age Groups: Children, Adults, and the Elderly

**DOI:** 10.3390/epigenomes8020013

**Published:** 2024-03-31

**Authors:** Teerachai Amnuaylojaroen, Nichapa Parasin

**Affiliations:** 1 School of Energy and Environment, University of Phayao, Phayao 56000, Thailand; 2 Atmospheric Pollution and Climate Research Unit, School of Energy and Environment, University of Phayao, Phayao 56000, Thailand; 3 School of Allied Health Science, University of Phayao, Phayao 56000, Thailand; nichapa.pa@up.ac.th

**Keywords:** PM_2.5_, toxicity, pathogenesis, health impacts, fine particulate matter

## Abstract

The effects of PM_2.5_ on human health fluctuate greatly among various age groups, influenced by a range of physiological and immunological reactions. This paper compares the pathogenesis of the disease caused by PM_2.5_ in people of different ages, focusing on how children, adults, and the elderly are each susceptible to it because of differences in their bodies. Regarding children, exposure to PM_2.5_ is linked to many negative consequences. These factors consist of inflammation, oxidative stress, and respiratory problems, which might worsen pre-existing conditions and potentially cause neurotoxicity and developmental issues. Epigenetic changes can affect the immune system and make people more likely to get respiratory diseases. On the other hand, exposures during pregnancy can change how the cardiovascular and central nervous systems develop. In adults, the inhalation of PM_2.5_ is associated with a wide range of health problems. These include respiratory difficulties, reduced pulmonary function, and an increased susceptibility to illnesses such as asthma, chronic obstructive pulmonary disease (COPD), and lung cancer. In addition, exposure to PM_2.5_ induces systemic inflammation, cardiovascular diseases, insulin resistance, and neurotoxic consequences. Evident disturbances in the immune system and cognitive function demonstrate the broad impact of PM_2.5_. The elderly population is prone to developing respiratory and cardiovascular difficulties, which worsen their pre-existing health issues and raise the risk of cognitive decline and neurological illnesses. Having additional medical conditions, such as peptic ulcer disease, significantly increases the likelihood of being admitted to hospital.

## 1. Introduction

The global deterioration in air quality can be attributed to the rapid processes of industrialization and urbanization [1]. PM_2.5_, which refers to fine particulate matter with a diameter of 2.5 μm or smaller, is regarded as one of the most problematic constituents of air pollution. It is a complex assemblage of particles with diverse chemical compositions, derived from a range of origins, including combustion, vehicle exhaust, manufacturing processes, and environmental events. According to a study conducted by Amnuaylojaroen and Parasin [2], prolonged exposure to PM_2.5_ was associated with a total of 4.14 million fatalities and 118 million lost disability-adjusted life years (DALYs). The composition of PM includes various toxic constituents that are soluble in water, primarily metals that have high dissolution and accessibility, as well as polycyclic aromatic hydrocarbons (PAH) that have specific chemical compositions [3]. On the other hand, the organic extract consists primarily of PAH with high oxidation potential, while the carbon essential component is primarily made up of metals that have low solubility and bioavailability [3]. The prolonged suspension of PM_2.5_ in the atmosphere is facilitated by its diminutive particle size, which allows for its inhalation and subsequent deposition within the respiratory system. The PM_2.5_ particles that have been deposited have the potential to penetrate the blood–brain barrier and subsequently distribute throughout the body via the bloodstream, exerting detrimental effects on multiple organs and systems and leading to adverse health outcomes [4,5,6,7].

Individuals who are exposed to increased concentrations of PM_2.5_ are more susceptible to the development of respiratory and cardiovascular ailments, as well as experiencing a decline in lung function and encountering other systemic health consequences [4,8,9]. Multiple studies have demonstrated that particulate matter (PM) has the potential to elicit both immediate and long-term effects on human health, resulting in detrimental effects on various physiological systems and organs, such as the respiratory, cardiovascular, digestive, nervous, immune, and reproductive systems [10,11,12,13,14]. The results of a prospective mortality study indicate that prolonged exposure to PM_2.5_ is a significant risk factor for mortality associated with lung cancer and cardiopulmonary disease. The research conducted by Pope et al. [9] demonstrates that there is a positive correlation between the levels of PM_2.5_ and the risk of mortality associated with lung cancer and cardiopulmonary disease. Additionally, a study confirmed the link between the oxidation potential of particulate matter (PM) components such as heavy metals, organic carbon, and elemental carbon and their negative effects on human health. Sisani et al. [15] investigate the toxicological mechanism by which particulate matter (PM) intensifies; there have been revisions to previous understandings in this field. The study revealed that even when exposed to low concentrations of PM_2.5_, there were negative impacts on human health. Furthermore, Shin et al. [16] conducted a study in which they observed a correlation between exposure to PM_2.5_ at an annual average concentration of 9.8 μg/m^3^ and a higher risk of heart attack and stroke.

Many studies have conclusively demonstrated the adverse impacts of exposure to PM_2.5_ on respiration and heart disease well-being. However, it is crucial to acknowledge the variability of PM_2.5_ impacts among different age cohorts to design specific interventions and health promotion strategies that are more effective. Children, due to their growing lung function and distinct vulnerabilities, exhibit heightened susceptibility to the detrimental consequences associated with exposure to PM_2.5_. Research findings have indicated that there is a correlation between exposure to PM_2.5_ during childhood and an elevated likelihood of experiencing respiratory symptoms, diminished lung function, and a higher incidence of asthma [17]. Moreover, recent research indicates that there may be neurodevelopmental consequences associated with exposure to PM_2.5_ in children, such as cognitive impairments and behavioral issues [18]. Gaining a comprehensive understanding of the mechanisms that drive these health impacts is of the utmost importance to effectively implement focused interventions aimed at safeguarding children’s respiratory health and enhancing their cognitive development. In the adult population, the health consequences associated with PM_2.5_ exposure are diverse, encompassing a wide range of respiratory and cardiovascular effects. Several epidemiological studies have established a correlation between prolonged exposure to PM_2.5_ and an elevated likelihood of developing chronic obstructive pulmonary disease (COPD), lung cancer, and respiratory infections [19]. Furthermore, there is a documented correlation between exposure to PM_2.5_ and an increased likelihood of experiencing cardiovascular events such as strokes, heart attacks, and hypertension [20]. The health effects observed in adults are attributed to various mechanisms, including chronic inflammation, oxidative stress, endothelial dysfunction, and systemic inflammation [4]. It is very important to understand the unique vulnerabilities and mechanisms that affect different age groups to come up with effective ways to reduce the health risks that come with adult exposure to PM_2.5_. The elderly demographic is characterized by heightened vulnerability due to age-related physiological alterations and pre-existing medical conditions. Numerous studies have consistently provided evidence indicating that exposure to PM_2.5_ in the elderly population is linked to elevated mortality rates, worsened respiratory diseases, occurrences of cardiovascular events, and cognitive deterioration [21]. The systemic impact on various organ systems, oxidative stress, and inflammation are just a few examples of the factors that can mechanistically explain the health effects mentioned [22]. Gaining a comprehensive understanding of the distinct vulnerabilities and fundamental mechanisms is of the utmost importance to formulate precise interventions aimed at safeguarding the health and overall welfare of the elderly demographic.

This research aims to provide insights by comparing the disease burden of PM_2.5_ among various age groups, emphasizing distinct susceptibilities and reactions resulting from physiological disparities. The results of this study will make a valuable contribution to the advancement of evidence-based interventions, regulatory actions, and focused policies aimed at addressing the specific vulnerabilities and health hazards associated with exposure to PM_2.5_ particles among different age groups.

## 2. Search Strategies

**Figure 1:**
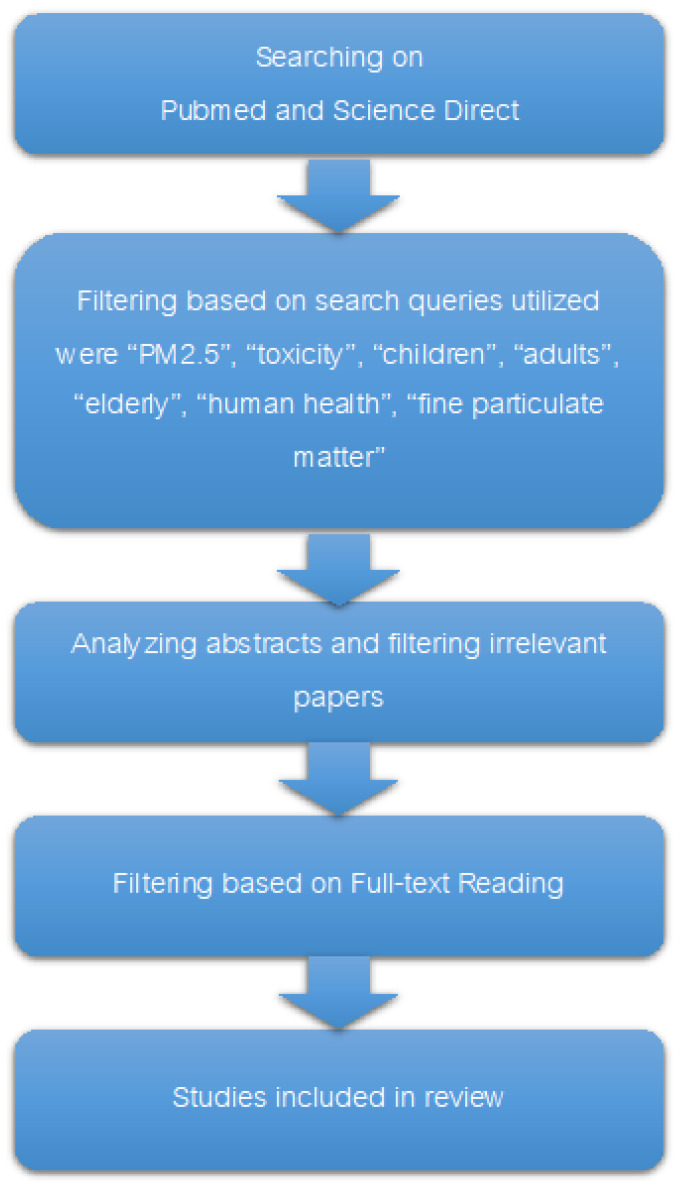
Diagram of the process of conducting a comprehensive search.

The literature review presented in this study was carried out with an approach to including a wide range of research articles, covering both clinical and experimental studies. As depicted in Figure 1, a detailed search was executed across the ScienceDirect and PubMed databases. The search strategy was carefully thought out to include a wide range of keywords, such as PM_2.5_, toxicity, children, adults, the elderly, human health, and fine particulate matter. These keywords were linked by search operators such as AND, OR, *, and parentheses to make sure that the retrieval process was both broad and specific. During the search, a total of 1451 articles were initially identified. Following the removal of duplicate submissions, a total of 10 articles were screened. After that, abstracts and titles were screened to exclude articles not directly relevant to the health impacts of PM_2.5_. Following this preliminary filtering, 36 articles were subjected to a thorough full-text review. To be included, the study had to directly look at PM_2.5_ and its effects on health. Studies that were not looking at the direct health effects of human exposure to PM_2.5_ or that were not focusing on the specific population groups were left out. After this stage, 14 studies were included in the main final review comparing the PM_2.5_ disease burden across age groups. The publication period of the articles considered ranged from 2000 to 2023, providing a comprehensive overview of the current understanding of PM_2.5_ health impacts. Articles were excluded primarily due to reasons such as a lack of direct relevance to the health effects of PM_2.5_, the absence of original research data (e.g., commentaries, editorials), or because they focused on non-human studies. This process of excluding studies made sure that the review focused on the most relevant and high-quality studies. This gave a strong summary of the evidence linking PM_2.5_ exposure to health outcomes in children, adults, and the elderly.

## 3. Characteristics and Sources of PM_2.5_

**Figure 2:**
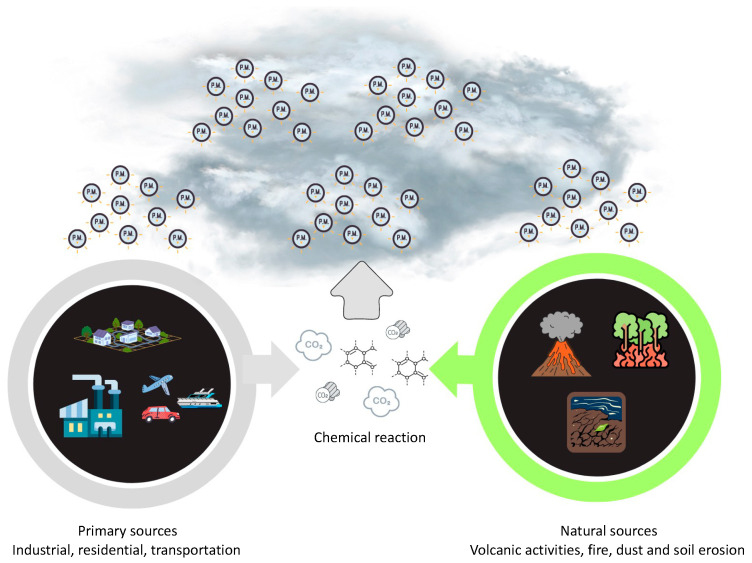
Emissions source of PM_2.5_.

PM_2.5_ refers to fine particulate matter that has a diameter of less than 2.5 μm. It possesses noticeable physical characteristics that impact its behavior and its impact on human health [23]. PM_2.5_ particles, due to their small size, could remain suspended in the atmosphere for prolonged durations, thereby enabling their penetration into the respiratory system upon inhalation [23]. As depicted in Figure 2, the levels of PM_2.5_ are susceptible to variation based on geographical location due to the influence of multiple sources, such as vehicle traffic, dust resuspension, biomass burning, power plants, sea salt, industrial emissions, ship emissions, and aviation emissions [24]. The morphology of PM_2.5_ particles has a significant impact on their behavior in the atmosphere and their deposition patterns within the respiratory tract [25]. It is possible for toxic compounds to stick to this bigger surface area because it makes it easier for it to interact with chemical species in the air [26]. The chemical composition of PM_2.5_ encompasses primary particles that are emitted directly into the atmosphere, including combustion byproducts, dust, and pollen. Additionally, secondary particles are formed through chemical reactions involving gases such as sulfur dioxide and nitrogen oxides [27]. Due to the potential for various chemical species to interact with biological systems and cause negative health effects, the specific composition of PM_2.5_ particles has a significant impact on their toxicity [26]. Its primary sources are the combustion and emissions resulting from vehicles powered by fuel, as well as the degradation of automotive components [28]. The primary constituents found in PM_2.5_ consist of black carbon, polycyclic aromatic hydrocarbons (PAHs), aryl hydrocarbons, volatile organic hydrocarbons, heavy metals, organic compounds, minerals, inorganic ions, and biological materials [29,30,31,32,33,34,35,36]. Collectively, these components account for a minimum of 79–85% of the total mass [37]. According to earlier research by Bangar et al. [37], PM_2.5_ has higher amounts of several elements, including aluminum (Al), arsenic (As), bromine (Br), calcium (Ca), chlorine (Cl), chromium (Cr), francium (Fr), potassium (K), magnesium (Mg), manganese (Mn), sodium (Na), lead (Pb), titanium (Ti), and zinc (Zn). Additionally, the presence of sulfate, nitrate, and ammonium ions has also been observed in these particulate matter samples.

## 4. Death Due to Particulate Matter in Different Regions

**Figure 3:**
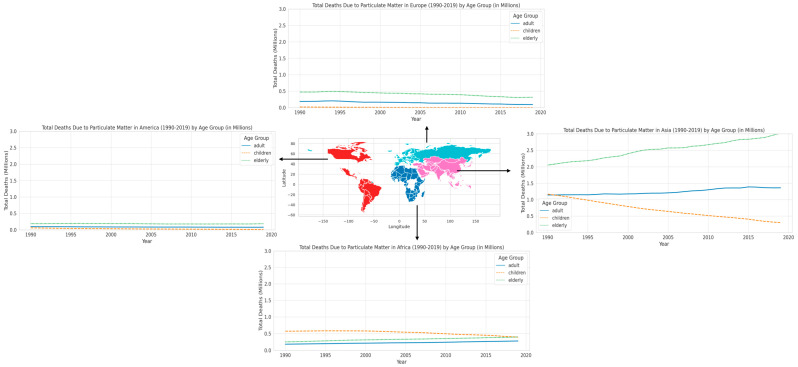
Total death in various age group due to particulate matter in different region during 1990–2019.

After examining the various traits and origins of PM_2.5_, we now comprehend the intricate nature of this fine particulate matter and its widespread existence in the environment. In the subsequent section, we shift our attention to the epidemiological findings, emphasizing how these attributes of PM_2.5_ result in noticeable and heterogeneous health outcomes in distinct geographical areas and age groups. This section demonstrates the substantial and diverse influence of PM_2.5_ on different age groups anfd regions, emphasizing a crucial public health issue. Regarding the patterns of mortality that have been identified, including the increased susceptibility of elderly people and the significant differences in regions like Asia and Africa, Figure 3 displays the total number of deaths in different age groups caused by particulate matter in various regions from 1990 to 2019, as reported by the Global Burden of Disease (GBD) [38]. Across all regions, a consistent trend is observed in terms of age groups: the elderly are the most impacted by PM_2.5_, followed by adults, while children experience the least amount of damage. The cause will be addressed in the following section. The disparity is especially pronounced in Asia, where the mortality rate among the elderly is notably higher compared to other regions. This highlights the profound health consequences of air pollution in this location. In Africa, although the old population also has a significant burden, and the mortality rate among children is significantly higher in comparison to Europe and America. This suggests the presence of regional differences and a widespread impact across all age cohorts. Asia has the highest mortality rate among all age categories when compared to other regions, highlighting the significant public health issues caused by air pollution. Africa exhibits significant repercussions for both children and the elderly, underscoring the extensive ramifications of PM_2.5_. The data from Europe and America consistently demonstrate that the older population is the most impacted. Nevertheless, the overall effect in these regions is less significant in comparison to Asia and Africa. The examination of temporal patterns indicates a progressive rise in fatalities attributed to PM_2.5_, particularly prominent in Asia and Africa. This pattern indicates a deterioration in air quality and its cumulative effect on health over time. The rise in mortality rates in Europe and America is less pronounced; nevertheless, the enduring effect on the senior demographic is still apparent.

Asia, especially South and East Asian countries, is notorious for having some of the highest levels of air pollution globally. Metropolises such as Delhi, Beijing, and Dhaka often record PM_2.5_ levels that greatly surpass the recommendations set by the World Health Organization [39]. The main factors contributing to this include primarily growing industrialization, urbanization, and increased dependence on coal and biomass for energy [40]. Several Asian countries exhibit high population densities, particularly in metropolitan regions. Greater exposure to PM_2.5_ is a result of the combination of high population density and urban pollution sources [41]. Urbanization also leads to lifestyle changes that can worsen the health effects of air pollution. Furthermore, in specific regions of Asia, agricultural activities such as crop burning make a substantial contribution to air pollution. Seasonal burning emits significant quantities of particulate particles into the atmosphere [42]. Additionally, variations in healthcare systems and access to care among various Asian countries may have an impact on the number of fatalities attributable to air pollution. In areas with underdeveloped healthcare systems, the effects of PM_2.5_ may be more noticeable [43]. Socioeconomic issues also contribute to the situation. Across many regions in Asia, a greater percentage of the populace is involved in outdoor manual work, while there is limited availability of air quality data and safeguards [44]. The efficacy of environmental regulations and air quality management programs differs among Asian nations, influencing the levels of PM_2.5_ and public health [45]. These findings emphasize the immediate necessity for focused actions, especially in areas with greater burdens such as Asia and Africa, from a public health standpoint. The diverse effects observed in different age groups underscore the significance of developing targeted solutions tailored to certain age groups to alleviate the health consequences of PM_2.5_. The ongoing and escalating influence of PM_2.5_’s consequences, particularly on the senior population, necessitates prompt implementation of air quality regulations and healthcare policies. This highlights the importance of adopting both proactive and reactive health strategies to address the adverse impacts of air pollution.

## 5. The Pathogenesis of PM_2.5_-Related Disorders in Various Age Group

As we proceed with investigating the different numbers of deaths caused by PM_2.5_ exposure in different areas, it is crucial to investigate the underlying pathophysiological mechanisms. This section delves into the pathophysiology of PM_2.5_-related illnesses. This investigation is critical for understanding the varying susceptibilities and responses to PM_2.5_ exposure. This investigation involved the classification of individuals into three distinct groups: children, adults, and the elderly. According to the guidelines provided by the National Institute on Aging (https://www.nih.gov/nih-style-guide/age, accessed on 13 March 2023), the term “children” generally encompasses individuals in the initial phases of growth and maturation, spanning from infancy to adolescence. The age range of humans normally extends from newborn to 18 years of age, with potential variations in certain circumstances [46]. Adults are typically individuals who have attained both physical and legal maturity and are regarded as fully developed [46]. The assumption of adult responsibilities—including employment, interpersonal relationships, and the use of decision-making skills—distinguishes this stage of human development [46]. The age range denoting the transition into adulthood exhibits variation across diverse societies, although it is commonly recognized to commence at approximately 18 or 21 years of age [47]. The term “elderly” generally pertains to individuals who have attained an advanced age, commonly linked to retirement and the later phases of life [48]. The age range in question exhibits some variability, but it typically encompasses individuals who are 60 or 65 years of age or older [48].

**Figure 4:**
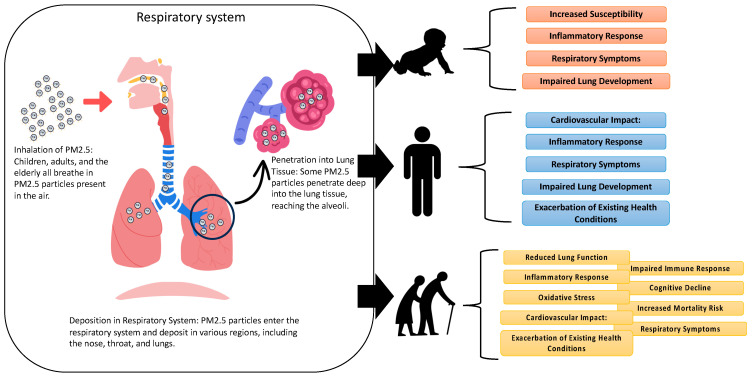
Mechanisms of toxicity of PM_2.5_ in children, adults, and the elderly.

Figure 4 delineates the mechanisms by which PM_2.5_ exerts its toxic effects on diverse age groups: children, adults, and the elderly. While the inhalation and deposition patterns of PM_2.5_ in the respiratory system are generally consistent across these groups, the resultant systemic impact and health implications are distinctly varied. In children, the deposition of PM_2.5_ in pediatric respiratory systems triggers acute inflammatory reactions, predisposing children to respiratory symptoms and increasing susceptibility to infectious diseases. Critically, these particles can impede lung development during crucial growth phases, leading to long-term respiratory deficits [49]. However, due to the immature blood–brain barrier in children, PM_2.5_ exposure may contribute to neurological developmental challenges and cognitive impairments [50]. Meanwhile, adults exposed to PM_2.5_ experience an array of effects, including respiratory symptoms, systemic inflammation, and heightened cardiovascular disease risks [50]. This exposure exacerbates pre-existing conditions like asthma or chronic obstructive pulmonary disease (COPD), impairs lung function, and can induce or worsen cardiovascular problems due to inflammatory and oxidative stress responses [51]. The potential for systemic effects, including metabolic and neurological disturbances, is also of concern. While the elderly are particularly vulnerable to PM_2.5_, exposure leads to pronounced inflammatory and oxidative stress responses [52]. This can exacerbate existing cardiovascular and respiratory conditions, compromise immune function, and potentially impact cognitive health, increasing the risk of neurodegenerative disorders [52]. In this group, PM_2.5_ exposure is closely linked to elevated mortality rates from cardiovascular and respiratory diseases.

### 5.1. Children

The pathogenesis mechanism of PM_2.5_ in children entails complex biological processes that have a substantial impact on their respiratory health and overall development. Children, since their respiratory systems are still developing, are especially vulnerable to the harmful impacts of PM_2.5_, which contains dangerous compounds such as heavy metals and polycyclic aromatic hydrocarbons (PAHs). These components could induce inflammation and oxidative stress in the respiratory system, resulting in airway inflammation, the increased production of reactive oxygen species (ROS), and the activation of pro-inflammatory pathways [49]. Children have respiratory systems that are more porous and have a higher relative ventilation rate than adults. Additionally, their immune system is less mature, making it less effective at detoxifying and expelling inhaled pollutants [53]. PM_2.5_ contains heavy metals, including arsenic, cadmium, and lead, which can deeply penetrate lung tissue and enter the circulation. These metals can cause significant harm, especially during the early phases, by interfering with cellular processes and DNA [54]. Oxidative stress is a crucial process through which PM_2.5_ exerts its impact. It involves an imbalance between the generation of reactive oxygen species (ROS) and the body’s capacity to neutralize these reactive substances or fix the consequent harm. PM_2.5_ induces an immunological response in the respiratory system, resulting in persistent inflammation, which increases the likelihood of developing respiratory conditions like asthma and impairs the growth of lung function [55]. The presence of heavy metals in PM_2.5_ leads to oxidative stress by producing reactive oxygen species (ROS) through Fenton-type processes and by reducing the levels of antioxidants. This results in oxidative damage to lipids, proteins, and DNA [52]. Polycyclic aromatic hydrocarbons (PAHs), which are present in PM_2.5_, could attach to DNA and create adducts. This might potentially result in genetic mutations. Additionally, PAHs can activate aryl hydrocarbon receptors (AhRs), which can cause alterations in gene expression related to inflammation and oxidative stress [56].

In addition, extended exposure to PM_2.5_ can worsen respiratory conditions due to its extremely small size, which allows it to settle in the lower airways. This leads to increased resistance in the airways and impaired lung function, causing symptoms such as coughing, wheezing, and difficulty breathing [54]. Significantly, there is an increasing body of research indicating that exposure to PM_2.5_ might cause neurotoxicity in young children. Block and Calderón-Garcidueñas [52] indicate that PM_2.5_ particles could cross the blood–brain barrier, causing neuroinflammation, oxidative stress, and anomalies in neurodevelopment. This can potentially result in cognitive impairments and a higher likelihood of neurobehavioral disorders. Furthermore, exposure to PM_2.5_ has been linked to changes in the epigenome of children, specifically affecting DNA methylation and histone modifications. This, in turn, has an impact on immunological responses, lung development, and the likelihood of developing respiratory disorders [57]. Prenatal and perinatal exposure to PM_2.5_ also presents substantial hazards. Research conducted by Brook et al. [4] and Kannan et al. [52] suggests that exposure to PM_2.5_ might lead to oxidative stress, systemic inflammation, and blood clotting, which can have negative effects on the health of a growing baby. The study by Feng et al. [58] and Valentino et al. [59] revealed that exposure to PM_2.5_ can cause changes in the function and structure of the placenta, which can negatively impact the development and growth of the fetus. Wick et al. [60] showed that PM_2.5_ particles can cross cellular membranes, indicating that tiny particulate matter that penetrates the placental barrier could have negative effects on the developing baby as its immune system continues to mature [61]. Exposure to PM_2.5_ during the prenatal period can significantly damage the critical developmental processes necessary for supporting life. This might potentially affect the development of the cardiovascular and central nervous systems, as well as interfere with lung maturation. This highlights the possible connection between exposure to PM_2.5_ during pregnancy and the death of infants. The severity of the negative effects during the time around birth can vary depending on the duration of exposure and the vulnerability of the fetus [62]. Exposure to PM_2.5_ during the early stages of pregnancy could lead to severe impairments in cognitive function, while later-stage exposure may be linked to deficiencies [63].

### 5.2. Adult

The pathogenesis mechanism of PM_2.5_ in adults encompasses intricate biological mechanisms that affect many organ systems and result in a range of health complications. When people breathe in PM_2.5_, the combination of heavy metals, organic chemicals, and combustion by-products included in these small particles deeply enters the respiratory system, reaching the alveoli, which are the small air sacs in the lungs responsible for gas exchange. The alveoli are highly susceptible to damage because of their fragile nature and their involvement in the transport of oxygen to the circulation. When PM_2.5_ particles reach the alveolar regions, they might trigger inflammation and oxidative stress in that specific area. Inflammatory cells are drawn to the site of injury, producing a number of cytokines and chemokines that contribute to the inflammatory response [64]. Oxidative stress arises from an overabundance of reactive oxygen species (ROS), which can overpower the body’s antioxidant mechanisms. This imbalance can result in cellular harm in the respiratory system and is a crucial element in the development of diseases like asthma, which exhibits airway hyperresponsiveness, and chronic obstructive pulmonary disease (COPD), which is characterized by a gradual restriction of airflow [56]. Moreover, the presence of toxic heavy metals such as arsenic and cadmium in PM_2.5_ might interfere with cellular and molecular mechanisms, ultimately resulting in the development of cancer. Prolonged exposure to these particles significantly raises the likelihood of getting lung cancer. Research indicates that PM_2.5_ can function as a comprehensive carcinogen, capable of both initiating and stimulating the growth of cancer cells [65]. Furthermore, the constituents of PM_2.5_ can be assimilated into the bloodstream and disseminated to other organs, potentially inducing systemic effects that extend beyond the respiratory system. PM_2.5_ exposure has been associated with elevated risks of cardiovascular disorders, such as hypertension and ischemic heart disease. This is because the particles can cause vascular inflammation, atherosclerosis, and disrupt cardiac autonomic function [4].

The study conducted by Thangavel et al. [66] demonstrates that PM_2.5_ particles could infiltrate the bloodstream, spreading throughout the entire body and resulting in systemic repercussions. Rajagopalan et al. [67] discovered that exposure to PM_2.5_ results in systemic inflammation and oxidative stress, which in turn leads to endothelial dysfunction and increases the vulnerability to cardiovascular illnesses. The induction of oxidative stress pathways involves Toll-like receptors and nucleotide binding receptors, resulting in the generation of reactive oxygen species (ROS) and the subsequent stimulation of inflammatory pathways [68,69,70]. The occurrence of oxidative stress can trigger the activation of MAPK pathways, NF-κB, and AP1, leading to inflammation, changes in membrane permeability, and the malfunction of mitochondria [71,72]. Exposure to PM_2.5_ also affects the cardiovascular system by causing alterations in the structure of the heart tissue and reducing its functionality, hence contributing to the development of cardiac disorders [73]. The systemic repercussions of this condition encompass inflammation, sympathetic activation, hypercoagulability, and endothelial dysfunction. These effects heighten the likelihood of ischemia events, arrhythmias, and heart failure [73]. Furthermore, PM_2.5_ has been found to be associated with insulin resistance and type 2 diabetes mellitus (T2DM). This is due to its impact on inflammation in visceral adipose tissue, the metabolism of lipids in the liver, and the consumption of glucose, as shown by Baccarelli et al. [74]. Upon inhalation, PM_2.5_ particles accumulate in the airways and lung cells, leading to oxidative stress, cellular malfunction, and apoptosis [75,76]. It could increase the production of Th2 cytokines and inflammatory mediators, which results in airway hyperresponsiveness and lung damage [77]. Xu et al. [78] found that exposure to PM_2.5_ negatively affects immunological functioning by altering the polarization of macrophages and the balance between Th1 and Th2 cells. This disruption contributes to the development of chronic bronchitis, COPD, and asthma [79,80]. Epidemiological studies have established a connection between PM_2.5_ and cardiovascular disorders such as arrhythmia and coronary artery disease [81,82,83]. Bhaskaran et al. [84] have specifically observed a correlation between exposure to PM_2.5_ and myocardial infarction. Moreover, PM_2.5_ particles could go to organs such as the liver and brain, which may result in neurotoxic consequences [85,86]. Epidemiological research indicates that air pollution has an influence on the central nervous system, with increased exposure to pollution being associated with a deterioration in cognitive function, feelings of depression, and negative effects on neuropsychological outcomes [87,88]. Calderón-Garcidueñas et al. [55] discovered increased levels of oxidative stress and indicators of neuroinflammation in people who were exposed to high levels of pollution. Guxens et al. [69] and Newman et al. [89] have documented that air pollution has neurotoxic effects, particularly on younger populations, and is associated with cognitive deficits and hyperactivity.

### 5.3. Elderly

Due to their increased sensitivity to air pollution because of age-related physiological changes and possibly pre-existing health conditions, the elderly are more vulnerable to its effects. Elderly individuals are more vulnerable to the detrimental impacts of inhaling PM_2.5_ particles on their respiratory systems. When the elderly inhale these small particles, the pollutants can go past the protective mechanisms of the upper airways and reach the sensitive alveolar regions, leading to direct harm [90]. The existence of these particles in the alveoli might result in an inflammatory reaction and oxidative stress, which play a crucial role in the development of chronic respiratory diseases such as chronic obstructive pulmonary disease (COPD) and pulmonary fibrosis. Moreover, PM_2.5_ has a broader influence that goes beyond the respiratory system, particularly impacting the cardiovascular well-being of older individuals. Studies have demonstrated that the small particles can move from one place to another inside the bloodstream, leading to many harmful effects throughout the body, such as inflammation and dysfunction of the inner lining of blood vessels. Endothelial dysfunction plays a crucial role in the advancement of atherosclerosis and can result in heightened arterial rigidity, diminished vascular responsiveness, and hypertension [91]. Exposure to PM_2.5_ has also been linked to irregularities in blood coagulation, which might worsen the likelihood of thrombotic events, including heart attacks and strokes [92]. The elderly population may experience a higher vulnerability to arrhythmias, possibly because of PM_2.5_’s impact on the autonomic nervous system. This might potentially lead to changes in heart rate variability and an elevated risk of fatal arrhythmias [93]. The impact of PM_2.5_ on the cardiovascular system is worsened in older individuals due to the natural loss in their physiological resilience associated with aging. This decline makes it more difficult for them to recover from the harmful effects of air pollution and increases the severity of the repercussions [94].

Furthermore, there is a correlation between exposure to PM_2.5_ and negative impacts on the brain health of older individuals. According to Block and Calderón-Garcidueñas [52], PM_2.5_ particles could travel to the brain, causing neuroinflammation, oxidative stress, and neurodegenerative alterations. These effects can potentially lead to cognitive decline, neurobehavioral disorders, and an increased vulnerability to conditions such as Alzheimer’s and Parkinson’s diseases [51]. A study conducted in Beijing found a notable rise in peptic ulcer blood loss in elderly adults after being exposed to high levels of PM_2.5_ for a short period of time [95]. The susceptibility in this age group may be attributed to a higher occurrence of comorbidities, intricate medication regimens, and an elevated frequency of NSAID-related peptic ulcer disease [96]. Wong et al. [97] discovered a direct link between extended exposure to PM_2.5_ and a rise in hospitalizations for peptic ulcer disease in the elderly. In addition, Knox [98] established a correlation between higher levels of air pollution and an increased mortality rate for peptic ulcer illness. Mutlu et al. [99] discovered that high levels of urban particulate matter (PM) exposure killed gastrointestinal epithelial cells, messed up tight junction proteins, and made the intestines more permeable. In addition, air pollution has the potential to induce both systemic and local inflammatory reactions [100], which play a key role in the development of pulmonary ulcerative bronchiolitis (PUB) [101]. There is a hypothesis suggesting that air pollution can contribute to the occurrence of PUB by disrupting the equilibrium of the gut microbiota, a condition known as dysbiosis [102].

## 6. Comparing the PM_2.5_ Disease Burden across Age Groups

This previous section has furnished an exhaustive comprehension of how PM_2.5_ impacts health distinctively in children, adults, and the elderly, emphasizing distinct susceptibilities and reactions to PM_2.5_. This section specifically examines the comparative disease burden associated with PM_2.5_ among different age groups. In children, inherent weaknesses due to physiologic growth processes make the respiratory system more susceptible to PM_2.5_ pollution. In children, the alveoli, which are the terminal structures in the respiratory system responsible for gas exchange, possess a substantial surface area in proportion to their body size. The broad alveolar surface is essential for efficient gas exchange and makes it highly susceptible to airborne contaminants. Children, since they have a bigger breathing capacity relative to their body weight, take in a greater amount of pollutants per kilogram of body weight compared to adults. This results in increased exposure and the accumulation of small particles in the alveolar region [56]. Once PM_2.5_ particles penetrate these lower parts of the lungs, they can bypass the usual protective systems that eliminate bigger particles. The exposure of alveoli to PM_2.5_ can trigger an inflammatory response marked by the influx of immune cells, including macrophages and neutrophils, into the lung tissues. The production of various inflammatory mediators, such as cytokines and chemokines, by these cells can lead to lung tissue damage and hinder respiratory performance [103]. Additionally, PM_2.5_ can cause oxidative stress, which is when the body makes too many free radicals (also called reactive oxygen species, ROS) and not enough antioxidants to fight them. Oxidative stress poses a significant threat to the growing lungs of children, as it can cause harm at the cellular and molecular level. This can result in various respiratory problems and perhaps hinder the normal growth and health of the lungs in the long run [52]. Exposure to PM_2.5_ in children can lead to a decrease in the growth of lung function, an increase in respiratory infections, the worsening of asthma symptoms, and a potential susceptibility to chronic lung disorders in the future. This is due to the combined impact of inflammation and oxidative stress [104].

In addition, the blood–brain barrier in children is incompletely developed, making it less efficient in blocking the entry of neurotoxic chemicals included in PM_2.5_ into the brain [105]. This can lead to neurological abnormalities and a higher susceptibility to developmental illnesses such as ADHD and autism spectrum disorders [104]. The blood–brain barrier (BBB) in children serves as a vital protective mechanism that controls the movement of chemicals between the bloodstream and the brain. In children’s developing brains, the blood–brain barrier (BBB) is not yet fully developed, which can diminish its ability to effectively block the entry of dangerous substances, such as neurotoxic compounds included in PM_2.5_, into the brain’s sensitive environment [105]. The underdeveloped state of the blood–brain barrier (BBB) in children means that the protective enzymatic and physical mechanisms are not yet fully operational. As a result, harmful substances such as heavy metals (lead, mercury), organic compounds, and ultrafine particles that make up PM_2.5_ can more easily pass into the brain compared to adults. Once these substances that are hazardous to the nervous system cross the blood–-brain barrier (BBB), they can directly harm the cells in the brain. This can result in oxidative stress and inflammation in the brain tissue, which has been linked to damage to the neurons and consequent neurological problems [106]. Increased exposure to neurotoxic chemicals during crucial stages of neurodevelopment can significantly impact neuronal plasticity and brain growth, potentially resulting in developmental problems. There is a growing body of research that establishes a connection between exposure to air pollution, particularly PM_2.5_, and a heightened susceptibility to neurodevelopmental disorders, such as attention deficit hyperactivity disorder (ADHD) and autism spectrum disorders (ASDs). Research has shown that children who are exposed to elevated amounts of air pollution tend to display impairments in memory, lower IQ scores, and behavioral issues that correspond to the symptoms of these disorders [107]. Furthermore, exposure to PM_2.5_ might cause systemic inflammation, which in turn can have additional impacts on the brain. Pro-inflammatory cytokines could pass through the blood–brain barrier (BBB) and worsen neuroinflammation, which may result in neurodegenerative alterations that might impact cognitive and behavioral outcomes in children [108].

Individuals who are adults and have pre-existing health conditions such as asthma or chronic obstructive pulmonary disease (COPD) are more likely to experience worsened symptoms when they encounter PM_2.5_ which refers to fine particulate matter that consists of different substances, including sulfates, nitrates, black carbon, and organic chemicals. These individuals have compromised pulmonary systems, making them particularly sensitive to the inflammatory and oxidative effects of PM_2.5_. Inhaling PM_2.5_ can trigger airway hyperresponsiveness in people with pre-existing respiratory problems. This condition causes the bronchial tubes to become excessively reactive to different stimuli, resulting in constriction and respiratory discomfort [109]. Occupational exposure to PM_2.5_ causes oxidative stress because the particles tend to make reactive oxygen species (ROS) in the respiratory tract. The ROS can cause damage to the airway epithelium, aggravating respiratory disorders like asthma, where an individual’s airways become irritated and restricted, leading to difficulties in breathing and increased mucus production [110]. Moreover, studies have demonstrated that PM_2.5_ particles play a role in diminishing lung function by stimulating inflammation, which in turn can cause alterations in the structure of lung tissue, resulting in decreased flexibility and functional ability [111]. This is especially harmful for patients with chronic obstructive pulmonary disease (COPD) as the disease’s progressive nature is marked by a pattern of obstruction that deteriorates as time goes on. Particulate matter can expedite the natural deterioration of lung function that occurs with age, especially in individuals with pre-existing pulmonary diseases [112].

PM_2.5_ has systemic effects on adults that go beyond the respiratory system due to its capacity to penetrate the bloodstream. This translocation leads to widespread inflammation, which is not limited to the lungs but affects several organs and systems in the body. Upon entering the circulatory system, PM_2.5_ particles can activate inflammatory pathways that have broad ramifications for the functioning of organs. An important consequence of this widespread inflammation is its effect on metabolic well-being. Studies have demonstrated that prolonged exposure to PM_2.5_ is linked to insulin resistance, which is a preliminary stage of type 2 diabetes [8]. Insulin resistance occurs when cells in muscles, adipose tissue, and the liver exhibit poor responsiveness to insulin, resulting in a reduced ability to absorb glucose from the bloodstream efficiently. PM_2.5_ particles cause widespread inflammation in the body, which can disrupt the pathways that regulate insulin. This disruption can result in higher levels of glucose in the blood, ultimately increasing the risk of developing type 2 diabetes. Moreover, there is a correlation between exposure to PM_2.5_ and reproductive health problems. Research has identified connections between air pollution and negative impacts on reproductive ability, the outcomes of pregnancy, and even the development of a fetus [113]. PM_2.5_ has the potential to impact reproductive health by causing endocrine disturbances or directly harming reproductive organs. Furthermore, the increasing concern lies in the carcinogenicity of PM_2.5_. The particles consist of diverse chemicals, including polycyclic aromatic hydrocarbons (PAHs) and heavy metals, which have been associated with an elevated susceptibility to cancer. Prolonged exposure to PM_2.5_ has been linked to an increased susceptibility to lung cancer and other types of cancer, primarily because it induces oxidative stress, DNA damage, and chronic inflammation [114].

The susceptibility of the elderly population to PM_2.5_ is significantly heightened because of age-related physiological alterations, such as decreased lung capacity and poor mucociliary clearance. The aging process is associated with a deterioration in the structural integrity and function of the lungs. This is marked by a loss in the ability of the lungs to recoil and changes in the way the chest wall moves, resulting in a decrease in lung capacity [115]. The inhalation of PM_2.5_, which could thoroughly permeate the respiratory system, exacerbates the natural loss in lung function. In older individuals, the mucociliary clearance system, which is responsible for eliminating inhaled particles and germs from the respiratory tract, also declines with age [116]. The cilia, which are little hair-like structures that coat the respiratory tract and exhibit synchronized movement to eliminate mucus and trapped particles, see a decline in their efficiency. This deficiency results in an extended accumulation of PM_2.5_ particles in the respiratory system, which in turn increases the duration of exposure and hence the likelihood of inflammation and harm to the tissues [117]. Exposure to PM_2.5_ can greatly worsen chronic respiratory disorders, including bronchitis and emphysema, which are more common in older individuals. Bronchitis is defined as the inflammation of the bronchial passages, resulting in heightened production of mucus and coughing. Emphysema, however, results in the impairment of the alveoli, hence decreasing the available surface area for gas exchange. The presence of PM_2.5_ in the lungs might worsen the symptoms of these diseases, resulting in heightened dyspnea, wheezing, and an overall deterioration in respiratory function [116]. Moreover, the weakened integrity of the blood–brain barrier in older individuals may allow neurotoxic PM_2.5_ components to enter, potentially leading to cognitive impairment and the advancement of neurodegenerative disorders [117]. Exposure to PM_2.5_ can cause systemic inflammation, which can worsen illnesses such as arthritis and kidney dysfunction. These conditions are more common in older adults.

## 7. Conclusions

The health effects of PM_2.5_ particles, which are characterized by their extremely small size and intricate composition, are significant and extensive. These particles, which can remain suspended in the air for long periods of time, could enter deeply into the respiratory system, leading to inflammation, oxidative stress, and the transportation of dangerous compounds. The toxicity of PM_2.5_ varies based on its precise chemical makeup, and its sources, both anthropogenic and natural, contribute to different exposure levels across locations. Exposure to PM_2.5_ in children is associated with a range of health problems, such as respiratory diseases, disruption of the immune system, and harmful effects on the nervous system. These effects are especially worrisome due to their potential to have lasting consequences for the health and development of children. Exposure to PM_2.5_ increases the susceptibility of adults to respiratory and cardiovascular diseases, metabolic disorders like diabetes, and neurological and cognitive impairments. This is particularly crucial for individuals with pre-existing diseases as PM_2.5_ can worsen health difficulties. PM_2.5_ exposure poses a significant risk to the senior population, leading to respiratory and cardiovascular issues, hastening cognitive decline, and causing gastrointestinal illnesses. Age-related physiological changes further increase the sensitivity of the elderly to the detrimental effects of PM_2.5_. Extended exposure among this population frequently results in heightened rates of hospitalizations and the worsening of preexisting health issues. Due to the significant threat that PM_2.5_ pollution poses to public health, it is crucial to enforce and enhance measures designed to decrease PM_2.5_ emissions. This involves coordinated efforts across sectors and geographies, stressing the establishment and implementation of legislation that cuts emissions, promotes cleaner technology, and raises public knowledge about air quality. To protect public health, it is imperative to consistently and collaboratively work towards reducing the effects of PM_2.5_ pollution, with particular attention to those who are most susceptible.

## Data Availability

All data generated or analyzed during this study are included in this published article.
